# Music and trauma: the relationship between music, personality, and coping style

**DOI:** 10.3389/fpsyg.2015.00977

**Published:** 2015-07-10

**Authors:** Sandra Garrido, Felicity A. Baker, Jane W. Davidson, Grace Moore, Steve Wasserman

**Affiliations:** ^1^Australian Research Council's Centre of Excellence for the History of Emotions, University of MelbourneMelbourne, VIC, Australia; ^2^Melbourne Conservatorium of Music, University of MelbourneMelbourne, VIC, Australia; ^3^Independent PsychotherapistHarrow, UK

**Keywords:** trauma, music therapy, arts therapies, coping style, individual differences

In a world that is dominated by news of conflict, violence and natural disasters affecting millions of people around the globe, there is a need for effective strategies for coping with trauma. The effects of such trauma on both individuals and communities, are deep and long-lasting (Sutton, 2002). Cultural techniques play an important role in helping communities to recover from trauma. Sports and games, for example, have been used in numerous settings with individuals suffering from post-traumatic stress disorder (Lawrence et al., 2010). Other arts-based therapies such as reading or creative writing are also proving to be effective means for dealing with the aftermath of traumatic events. Music can also play a role in helping individuals and communities to cope with trauma, whether it be through the intervention of music therapists, community music making programs or individual music listening. However, despite the abundance of positive examples of the value of the arts in trauma recovery, music, and the arts receives little recognition by leaders in global health issues (Clift et al., 2010). This paper will argue, therefore, that there is a need for a solid empirical evidence base that can illuminate the mechanisms by which music and arts therapies are effective, as well as consideration of how individual differences in personality and coping style can moderate participant responses to such therapies.

## Trauma and its effects

While the word “trauma” can refer to both physical and psychological trauma, in this paper we focus on the latter. However, defining psychological trauma is in itself problematic. The current *Diagnostic and Statistical Manual of Mental Disorders (DSM-V*, APA 2013), in the context of defining post-traumatic stress disorder (PTSD), defines a traumatic event as “actual or threatened death, serious injury or sexual violence,” whether personally experienced or witnessed, or experienced vicariously. This definition can include a variety of stressors of varying magnitude, frequency and duration. In addition, individual appraisal of an event can lead to differing levels of impact upon each person experiencing the event (Weathers and Keane, 2007). As described by Sutton (2002), however, most scholars agree that three aspects are central to an understanding of trauma: “shock, wound and a lasting effect” (p. 22).

Two categories of trauma related disorders are included in the DSM: acute stress disorder, which relates to the acute and immediate effects of a traumatic event, and PTSD, a more chronic and long-lasting condition. However, many people may suffer from the impact of a traumatic event at sub-threshold levels. Among the disturbing effects at both clinical and sub-clinical levels of trauma may be emotional numbing, re-experiencing the event, survivor guilt and feelings of responsibility, anger, and heightened arousal levels (Honig et al., 1999). Trauma has also been found to have a detrimental effect on attention, memory and processing speed in children, as well as skewing their expectations of safety and security in the future (Pynoos et al., 2007).

## Music and the arts in coping with trauma

Over the past decade, music therapy has emerged as a creative art form that has been used to address stress and coping with survivors of trauma. In a case study of 8–11 year old children who had survived a tornado in the Southeastern United States, for example, music was used to assist the children in expressing feelings and to help them make the transition back to school (Davis, 2010). The children created a musical composition based on their feelings about the tornado, enabling them to acknowledge and process their emotions in a healthy and healing way.

McFerran and Teggelove (2011) similarly used music interventions with young people after the “Black Saturday” fires in Victoria, Australia in 2009. Improvisation, songwriting and the sharing and discussing of songs was used within the program, and participants indicated that playing music with others who had been through similar experiences and who understood them, was important. The musical experiences were reported to have helped people to “hear one another,” bond with others also experiencing loss, and regain confidence.

Survivors of violence have also benefited from participation in music therapy programs. In one project working with survivors of the September 11 attacks on the World Trade Center in New York City (American Music Therapy Association, 2011), 33 music therapists provided over 7000 programs to children, adults and families. The programs were designed to reduce stress, improve coping, and process the trauma associated with the crisis by drawing on a range of techniques including musical improvisation, songwriting, singing, sharing stories, and relaxing with music.

Music has also been used as a healing agent in contexts that do not involve a trained music therapist, such as with survivors of the post-election violence in Kenya in 2007 (Akombo, 2009). In the study reported by Akombo, a community musician used music to recall and experience the trauma, incorporating humor into his work with survivors to help them deal with the distress associated with the initial violence as well as the resulting displacement. While little research has been conducted specifically in relation to trauma survivors outside of music therapy contexts, the literature indicates that various self-determined musical activities including listening, playing, singing, dancing and songwriting, are commonly used for coping and mood regulation among both adolescents and adults (Mayers, 1995; Shields, 2001; Miranda and Claes, 2009; Davidson and Fedele, 2011; Monteiro and Wall, 2011; Saarikallio, 2011).

There is also evidence that people benefit from other creative therapies such as reading and writing and it is important to consider how these therapeutic activities may be used both individually and alongside one another. The writer Arnold Zable has reported that survivors of the “Black Saturday” bushfires responded positively to creative writing workshops, with one participant commenting, “I'm finding this more powerful than counseling”^1^. Projects like “Get into Reading,” used in prison communities, hostels for the homeless, and in mental health care by the Mersey Care NHS Trust (Billington et al., 2013). Encourage groups to read aloud together. They are distinct from the “book group” model because of the performative nature of the reading experience. More solitary forms of reading can also offer comfort for many readers (Moore and Pyke, 2007).

Memorizing poetry, especially poems that are applicable in some way to the individual's situation (Petermann, 1996; Kelly, 2014), is also able to elicit non-linguistic biopsychosocial effects in a similar way to music therapy: providing a sense of safety, management of anxiety and emotional processing, which then serves as a foundation to the therapeutic goal of finding one's voice—a more coherent narrative—through which to process traumatic symptoms.

As noted in the studies cited above and others, music, and music therapy researchers argue that the benefits of musical activities include: mood improvement, self expression, catharsis, facilitating grieving, relaxation, reflection, socialization, community building, stress reduction, and more. As with compositional forms of music therapy, those involved in writing groups have reported that the process of writing about their trauma allows them to regain their agency, to tell their own stories. Reading and memorizing of texts can similarly allow one to express oneself and to relive and reflect on memories and experiences.

One might see expressive arts therapies as playing the part of a “resource replacement,” based on a conservation of resources model of stress (Freedy et al., 1992; Hobfoll, 2001). Here we recognize that the perception of a net loss of resources accompanying a traumatic event (objects, personal characteristics, conditions, or energies which have value to us) engender the destabilizing and overwhelming “black hole of trauma” (Bloom, 2010, p. 1). This sense of loss needs to be offset by other resources. If this cannot be done through direct replacement of the objects lost, then one might want to consider “symbolic replacement or replacement through indirect means” (Hobfoll, 1989, p. 518).

## Individual personality and coping style

While the benefits of the arts therapies discussed above are multiple, there is some evidence that people do not always respond in the same way to music or other creative arts in dealing with trauma. Baikie and McIlwain (2008), for example, found that expressive writing was more beneficial for individuals with high scores in alexythmymia and splitting, but less useful for people with a repressive coping style. Miranda and Claes (2009) found that adolescent boys who used music listening as an emotion-oriented coping strategy tended to have higher levels of depression, whereas girls who used music listening as a problem-oriented coping strategy tended to have lower depression levels. Similarly, Garrido and Schubert (2013, 2015) have found across several studies that people with high scores in rumination do not obtain the same psychological benefits from listening to sad music as people with more reflective personalities.

While the effectiveness of music therapy and other arts programs have been clearly demonstrated in the literature, and individual therapists are themselves highly aware of the way in which the programs affect participants as individuals, clear experimental evidence that demonstrates the mechanisms at work and the influence of differences in personality and coping style, is currently lacking. There is little understanding of which elements of these programs are crucial to their effectiveness. Do creative activities such as songwriting, for example, confer benefits superior to comparatively passive activities such as reading? Do group activities confer greater benefits than solitary activities? Are the lyrics of the music involved more important than the acoustic features? Or is the effect of music a synergy between these elements? Do the benefits of musical activities, in fact, outweigh those of other art therapies? Or, do the benefits of various types of engagement with the arts differ depending on various factors in relation to the individual involved?

The answers to these questions are crucial, given the increasing numbers of arts programs that are emerging in the wake of traumatic events around the world. The establishment of an experimental evidence base for understanding the factors involved would be beneficial to both the design of effective interventions and for influencing community health strategies and public health policy for dealing with the aftermath of trauma.

### Conflict of interest statement

The authors declare that the research was conducted in the absence of any commercial or financial relationships that could be construed as a potential conflict of interest.

## Footnotes

^1^http://www.culturaldiversity.com.au/news/94-news-and-events/214-the-wonder-of-words
